# Using a MDMA- and LSD-Group Therapy Model in Clinical Practice in Switzerland and Highlighting the Treatment of Trauma-Related Disorders

**DOI:** 10.3389/fpsyt.2022.863552

**Published:** 2022-04-25

**Authors:** Peter Oehen, Peter Gasser

**Affiliations:** ^1^Private Practice of Psychiatry and Psychotherapy, Biberist, Switzerland; ^2^Private Practice of Psychiatry and Psychotherapy, Solothurn, Switzerland

**Keywords:** MDMA, LSD, psychedelic-assisted psychotherapy, PTSD, C-PTSD, depression, psychedelic group therapy

## Abstract

The Swiss Federal Act on Narcotics allows for the restricted medical use of scheduled psychotropic drugs in cases of resistance to standard treatment, and preliminary evidence of efficacy of the scheduled drug for the particular condition. Since 2014, the authors have obtained 50 licenses on a case-by-case basis and developed a psychedelic-assisted group therapy model utilizing MDMA and LSD. The majority of the patients taking part in the psychedelic group therapy suffered from chronic complex post-traumatic stress disorder (c-PTSD), dissociative, and other post-traumatic disorders. Treatment modalities, typical developments and problems encountered during and after the psychedelic experiences are described. Recurrent depression poses a frequent problem, and requires special attention. Symptoms of c-PTSD predominantly addressed by the psychedelic experiences are the regulation of emotions and impulses, negative self-perception, alterations in relationships to others, as well as meaning, recall, and processing of traumatic memories. C-PTSD needs a larger number of psychedelic experiences in contrast to PTSD resulting from single trauma. In this model MDMA was most often used in the first phase to enhance motivation to change, strengthen the therapeutic alliance, allowing it to become more resilient, stress-relieved and less ambivalent. When emotional self-regulation, negative self-perception and structural dissociation had also begun to improve and trauma exposure was better tolerated, LSD was introduced to intensify and deepen the therapeutic process. The majority of participants improved by clinical judgement, and no serious adverse events occurred. A short case vignette describes a typical process. The experiences with this model can serve to further develop the method of psychedelic-assisted psychotherapy (PAP) and to give directions for future research.

## Introduction

Since the discovery of LSD by Albert Hofmann in 1943, there have been many attempts to prove the effectiveness of psychedelics such as LSD and psilocybin as adjuncts to psychotherapy. In the 1950's to the 1970's LSD was tested for treating anxiety, depression and pain in advanced stage cancer (1, 2), the treatment of addiction (3) and to enhance the effects of psychotherapy in general (4). Most of these studies do not meet modern research standards and are therefore of limited validity. After a hiatus of several decades which began with the worldwide ban of psychedelics in the 1970's, clinical research into psychedelic-assisted psychotherapy has restarted with the publication of results from several small phase 2 trials applying rigorous methodology to examining psilocybin and LSD for depression, anxiety and depression related to advanced-stage cancer and life-threatening diseases. These have yielded promising results (5–9).

The entactogenic compound MDMA met a similar fate. After its introduction into psychotherapy in the late 1970's, when its anxiolytic and prosocial effects were used to treat trauma-related disorders, anxiety disorders and in couples therapy (10), it rapidly spread as a recreational drug which also led to its worldwide ban in 1985–86. After a phase of public and scientific concern of serious neurotoxic effects, which could be relativized in regard to the application of MDMA in controlled clinical situations (11), clinical research since 2010 has shown positive effects for MDMA-assisted psychotherapy for social anxiety with autistic adults (12) and especially for treatment-resistant post-traumatic stress disorder (PTSD) (13–15), the latter studies showing high effect sizes. A first large phase 3 study in North America has recently been published (16).

All of the above-mentioned studies used an individual setting with one or two sitters/therapists per patient. The only documented exceptions to the dominant single setting in psychedelic psychotherapy took place in Switzerland from 1988 to 1993, when a small number of psychiatrists in private practice held licenses for the application of MDMA and LSD in psychotherapy also using a group setting (17).

Another more recent exception is a study of psilocybin-assisted group psychotherapy for demoralized male long-term AIDS survivors (18). This study investigated safety and feasibility of one psilocybin session conducted in an individual setting as an adjunct to sessions of talk therapy in groups.

The differences in the model we present are:

a) Our patients participated within the framework of—on one hand—individual talk therapy and—on the other hand—group sessions in which all patients simultaneously took a psychedelic substance.
b) Psilocybin was used in the 2020 study as a one-time adjunct to conventional (group) psychotherapy, where we administered a psychedelic compound multiple times accompanied by integrative psychotherapy.
c) The psilocybin study had a rigorous scientific-methodological background examining a well-defined study population in contrast to our purely clinical setting with a mixed population.

The purpose of this article is to describe the application of MDMA and LSD as adjuncts and catalysers of psychotherapy in a mixed clinical population of patients who did not respond to standard treatment, using a group model, suggesting the feasibility and safety of this model and highlighting the characteristics and typical developments in the treatment of trauma-related disorders. Because of the regulatory limitations, research is not allowed so as to not circumvent regular drug development practice, and we therefore did not use standard psychometric scales regularly. Our starting point in 2014 was therefore a purely clinical situation without any research ambitions, resulting in only a very coarse, descriptive evaluation of the treatment results.

In the United States, and in some other countries, a similar program called “Expanded Access,” applied for by the Multidisciplinary Association for Psychedelic Studies (MAPS), was approved by the FDA in 2019. The program's purpose is much the same as the one for limited medical use in Switzerland, namely to grant access to potentially beneficial investigational treatments for people facing a serious or immediately life-threatening condition for which there is no satisfactory treatment currently available (19).

## Current Situation of Psychedelic-assisted Psychotherapy in Switzerland

The Swiss Federal Act on Narcotics and Psychotropic Substances, art. 8, para. 5, allows for the restricted medical use of scheduled psychotropic substances (also referred to as “compassionate use”), but requires that existing treatments are ineffective and that preliminary scientific evidence suggests the efficacy of a scheduled drug for the particular condition^1^. Licenses were issued case-by-case for the use of MDMA or LSD on the basis of a detailed and comprehensive application to the Federal Office of Public Health (FOPH) by the attending psychiatrist, which also includes a written informed consent by the patient.

After the Swiss MDMA/PTSD study and the Swiss LSD/anxiety in life-threatening disease study results were published in 2013 and 2014 (6, 15) the principal investigators of these two studies were able to obtain licenses from the Swiss FOPH for the administration of LSD and MDMA to patients from their own patient pools in their private practices. Since then, increasing public attention on the issue of psychedelics and psychedelic-assisted psychotherapy led to the referral of patients and requests for psychedelic-assisted treatment from patients from inside and outside the country. For legal reasons—licenses are issued solely for the treatment of Swiss residents—and for therapeutic and practical considerations (travel and insurance issues) only patients from Switzerland who were able to participate in the preparatory and integrative therapy before and after drug-assisted sessions were treated.

In a first phase beginning in 2014, MDMA or LSD was administered in an individual setting. From 2015, a psychedelic-assisted group therapy model was established. Over the course of time, patients predominantly with trauma-related disorders, especially complex post-traumatic stress disorder (c-PTSD) were admitted to the group. All patients had previously undergone multiple psychotherapeutic and psychopharmacological treatments and had failed to improve in regard to symptom reduction and general wellbeing and were therefore considered treatment-resistant.

A more detailed article on the history and nature of psychedelic-assisted group therapy was recently published by Gasser (20).

The collaboration between the authors ended in 2020 and the group was closed.

## Patients, Description and Focus on c-PTSD

### Patient Characteristics and Focus on c-PTSD

Due to the growing publicity regarding MDMA for PTSD, the focus of referrals and requests for therapy in the first author's practice shifted increasingly toward trauma-associated disorders. This led to a predominance of trauma-associated diagnoses in the presently described patient population. Furthermore, when we initiated the group, patients from the first author's practice were in the majority. These circumstances certainly influenced the general atmosphere and content-related orientation of the group, but a specific “tailoring” of the overall therapeutic approach was not necessary or intended. Also, we did not in any way limit the access to the group by favoring trauma-related disorders. As for the cluster headache/migraine subgroup (Table 1), all those involved quickly realized that the group setting did not meet the specific needs and goals of this subgroup.

**Table 1:** Patient characteristics and index diagnosis.

**Patient characteristics (2014–2020)**	PG^*^	PO^*^	**Total**
**Total individual licenses**	26 (52%)	24 (48%)	50 (100%)
**Gender**			
Female	16 (62%)	17 (71%)	33 (66%)
Male	10 (38%)	7 (29%)	17 (34%)
**Country of origin**			
Switzerland	23 (88%)	20 (83%)	43 (86%)
EU (D: 2; PT: 1; B: 1: NL: 1)	3 (12%)	4 (17%)	8 (14%)
**Age median**			
Range: 31–74y	48.5	45	-
**Marital status**			
Single	9 (35%)	10 (42%)	19 (38%)
Married/living with partner	14 (54%)	9 (38%)	23 (46%)
Divorced/separated	3 (11%)	5 (20%)	8 (16%)
**Work status**			
On disability	5 (19%)	7 (29%)	12 (24%)
Fit for limited employment	3 (12%)	3 (13%)	6 (12%)
Working full-time	12 (46%)	13 (54%)	25 (50%)
Retired	6 (23%)	1 (4%)	7 (14%)
**History of drug abuse**			
Alcohol	1 (4%)	4 (17%)	5 (10%)
Cannabis	3 (12%)	2 (8%)	5 (10%)
Cocaine	0 (0%)	1 (4%)	1 (2%)
Patients with previous recreational use of psychedelics (total reported <4 occasions with Ayahuasca, magic mushrooms, MDMA, and/or LSD)	5 (19%)	7 (29%)	12 (24%)
**Index diagnosis**			
Trauma-related disorders: PTSD/c-PTSD/Dissociative Disorder - with comorbid depression - with comorbid borderline personality traits - with comorbid personality disorder	6 (23%) - (5) - (1) - (1)	15 (63%) - (7) - (3) - (0)	21 (46%) - (12) - (4) - (1)
- Depression	4 (15%)	6 (25%)	10 (20%)
- Cluster headache/severe migraine	5 (19%)	0 (0%)	5 (10%)
- OCD	1 (4%)	1 (4%)	2 (4%)
- Anxiety disorders: General Anxiety Disorder (GAD), Anxiety Disorder in life- threatening disease	9 (35%)	1 (4%)	10 (20%)
- Autism Spectrum Disorder (ASD)	1 (4%)	1 (4%)	2 (4%)

* *PG, Peter Gasser; PO, Peter Oehen*.

We treated a total of 50 patients between 2015 and 2020. Table 1 presents more detailed demographic data. Concerning the index diagnoses, a total of 21 patients with trauma-related disorders was the largest subgroup, 12 of whom had comorbid depression, followed by 10 cases with depression, 10 cases with anxiety disorders, two cases with obsessive-compulsive disorder (OCD), and two cases with ASD.

### Inclusion Criteria

Treatment-resistance in the case of trauma-related disorders was defined as having had at least two unsuccessful courses of psychotherapy and at least one psychopharmacolo-gical treatment with an SSRI or SNRI. In the case of depression and anxiety disorders, treatment-resistance was defined as not having sufficiently responded to at least 2 antidepressants, augmentation strategy and psychotherapy.

All patients participating in psychedelic-assisted psychotherapy were required to be in regular psychotherapy with one of the authors. In exceptional circumstances, we treated patients with psychedelic-assisted psychotherapy who were under treatment of an external or referring therapist. In this case, patients were required to have a series of diagnostic and introductory sessions and at least one integrative psychotherapy session after each psychedelic-assisted experience with one of us. They were also required to agree to a professional exchange between all therapists involved in the treatment.

### Exclusion Criteria

Borderline personality disorder (BPD) as an index diagnosis was excluded. A history of psychotic episodes or bipolar disorder in patients and in first-degree relatives were considered as a contraindication and therefore motives for exclusion. Four cases with an index diagnosis of c-PTSD had comorbid borderline personality traits. These patients were carefully assessed in regard to the risk of self-harm, suicidality, impulsivity and the stability of the therapeutic relationship before being accepted for psychedelic-assisted psychotherapy. Patients with acute drug dependency were excluded. Cannabis consumption for medical reasons was accepted.

During the reporting period, we rejected a very large number of requests and referrals due to the above-mentioned exclusion criteria such as bipolar disorder, psychosis (borderline) personality disorder, but mainly due to limited capacities, requests from outside Switzerland, no prior standard treatments and young age (<20 y).

## Materials, Description, and Dose

### Substances

The MDMA and LSD were provided by the Pharmacological Department of the University Hospital of Basel, Switzerland, which was responsible for all handling, quality controlling and encapsulation. MDMA (3,4-methylendioxymethamphetamine-HCl) was administered in 100 and 25 mg capsules. LSD (Lysergic acid diethylamide-HCl) was supplied in drinking vials (dissolved in alcohol) 100 and 25 mcg. Until 2020 only LSD and MDMA were available, but since then psilocybin has also become an option.

### Choosing Substance and Dose

Psychotropic medications with the potential to interact with or which could dampen the effects of MDMA or LSD (antidepressants and neuroleptics) were discontinued at least five half-lives before the psychedelic-assisted sessions. The duration of tapering was 1–2 weeks, depending on the original dose level and the individual tolerance of rapid dose reduction.

All somatic medications such as antihypertensive drugs, thyroid preparations or anti-Parkinson's medication were continued.

MDMA was indicated primarily for the first phase of psychedelic-assisted psychotherapy because of its effects, which are therapeutically and subjectively easier to handle and not so overwhelming (in contrast to LSD), its anxiolytic and prosocial properties, as well as to enhance motivation to change, to strengthen the therapeutic alliance to become more resilient, stress-relieved and less ambivalent. Patients with severe trauma-related disorders particularly benefit from a gradual approach beginning with lower doses of MDMA (75–100 mg), which facilitates the building of trust and feeling of security and strengthening the resilience of the therapeutic alliance. The maximum dose of MDMA was 125 mg. In some rare cases, a booster dose of 50 mg was given after ~2 h in situations where the effects of MDMA were weak or very short.

LSD was indicated for later stages of the therapy when some trauma processing had already taken place and exposure to traumatic material could better be tolerated. In particular, negative self-beliefs deeply anchored in the personality responded only to LSD. Switching from MDMA to LSD was based on clinical judgement as well as the readiness of the patient and his or her trust in the therapeutic process to go deeper and allow for more confrontation.

If trauma was not the main focus, such as in OCD, depression or with end-of-life issues, LSD was administered from the outset. The dose of LSD ranged from 100 to 200 mcg, depending on the target symptoms, personality and psychological defense structures. Patients who were rigid, emotionally highly controlled and compulsive needed higher doses, whereas emotional instability, impulsiveness, and histrionic personality structure would benefit more from lower doses. In most cases, the optimal dosage of LSD was titrated over the first few sessions. In general, this dose range was found to be optimal for producing the desired drug effects while at same time allowing for participation in the group process.

## Psychedelic-assisted Group Psychotherapy Model

### Background

Our model is based on and further developed from one which was already used in Switzerland from 1988 to 93 (17). Our practical work is based on the assumption that many, if not most, psychological disorders originate in some kind of traumatization—abuse or neglect—usually beginning early in life, even perinatally or transgenerationally, leading to developmental constraints, pathologies, and/or specific trauma-related disorders. Our therapeutic psychotraumatological background is an exposure-based approach and includes the concept of structural dissociation (21). The theory of structural dissociation postulates that under the pressure of severe and repetitive trauma, the mental capacity to integrate such experiences is overstrained and the personality divides into different subparts, each with its own psychobiological characteristics and degree of dissociation. For the application of psychedelics, we follow the emerging “reset paradigm,” which postulates that the mystical-type peak experience occasioned by psychedelics correlates with therapeutic effects and is a psychological marker for the restoring of healthy network connectivity (22–24, 36).

In order to fully understand the effects of this therapy, the four perspectives of set, setting, substance and sitter/therapist have to be taken into account. In our view, psychedelic therapists should have experienced drug-induced altered states of consciousness in a therapeutic setting themselves in order to fully appreciate and understand the mechanisms of action, possibilities, limitations, and pitfalls of the process.

### Complex Post-traumatic Stress Disorder

Herman first proposed the concept of complex post-traumatic stress disorder (c-PTSD) in 1992. It describes in a coherent and integrative way the multifaceted symptoms of traumatized patients who suffered repeated or prolonged physical, sexual and/or emotional abuse and neglect during childhood and adolescence, often by close caregivers, where escape was not possible due to physical, developmental or environmental/social constraints (25). The very diverse clinical presentation of this common patient population in everyday clinical practice goes beyond the scope of the PTSD diagnosis as described in the DSM-5 and ICD-10. There is a considerable overlap with other trauma-related disorders, predominately borderline personality disorder (BPD). It has also been suggested that PTSD, c-PTSD, and BPD are a clinical and biological continuum of symptom severity to be classified as trauma-related disorders (26).

Despite the clinical usefulness of this concept for diagnosis, understanding and for formulating treatment guidelines, the concept of c-PTSD was for a long time discussed very controversially and was accordingly not included in the DSM-5 revision. In contrast, in the now implemented ICD-11, PTSD, and c-PTSD have been included as two distinct conditions under a general category “Disorders specifically associated with stress” based on preliminary evidence that c-PTSD is a common, more frequent and more debilitating condition than PTSD (27).

We followed the ISTSS guidelines for the treatment of c-PTSD that were valid at the time (28, 29), which defined the disorder by differentiating eight symptom clusters of which three include the core symptoms of PTSD namely re-experiencing, avoidance/numbing and hyperarousal. In addition, there are five clusters of symptoms associated with impaired self-regulatory capacities: (a) emotion regulation difficulties, (b) disturbances of relational capacities, (c) alterations in attention and consciousness (e.g., dissociation), (d) adversely affected belief systems, and (e) somatisation/somatic distress or disorganization.

The recommended model for treatment for c-PTSD (28, 29) at that time consisted of three phases of treatment. The first was referred to as the stabilization and skills strengthening phase, focusing on ensuring safety (e.g., stopping exposure to perpetrator/trauma, reducing self-destructive behavior), reducing symptoms and increasing basic psychosocial competences. The second phase focused on trauma processing and integration, and the third phase consisted of the transition of treatment gains to everyday life i.e., social life and relationships and in the educational/work field.

The common key components of the major existing and empirically supported, effective trauma-focused psychotherapies are: (a) Psychoeducation on the nature and course of psycho-physiological reactions to trauma, how to cope with intrusive memories and management of distress. (b) Emotion regulation and coping skills. (c) Exposure to traumatic memories. (d) Cognitive processing and restructuring and/or meaning making. (e) Targeting emotions such as fear, guilt, shame, anger, or grief and sadness. (f) Reorganization of memory functions and creation of a coherent trauma narrative (30).

### Preparatory phase

**Figure 1:**
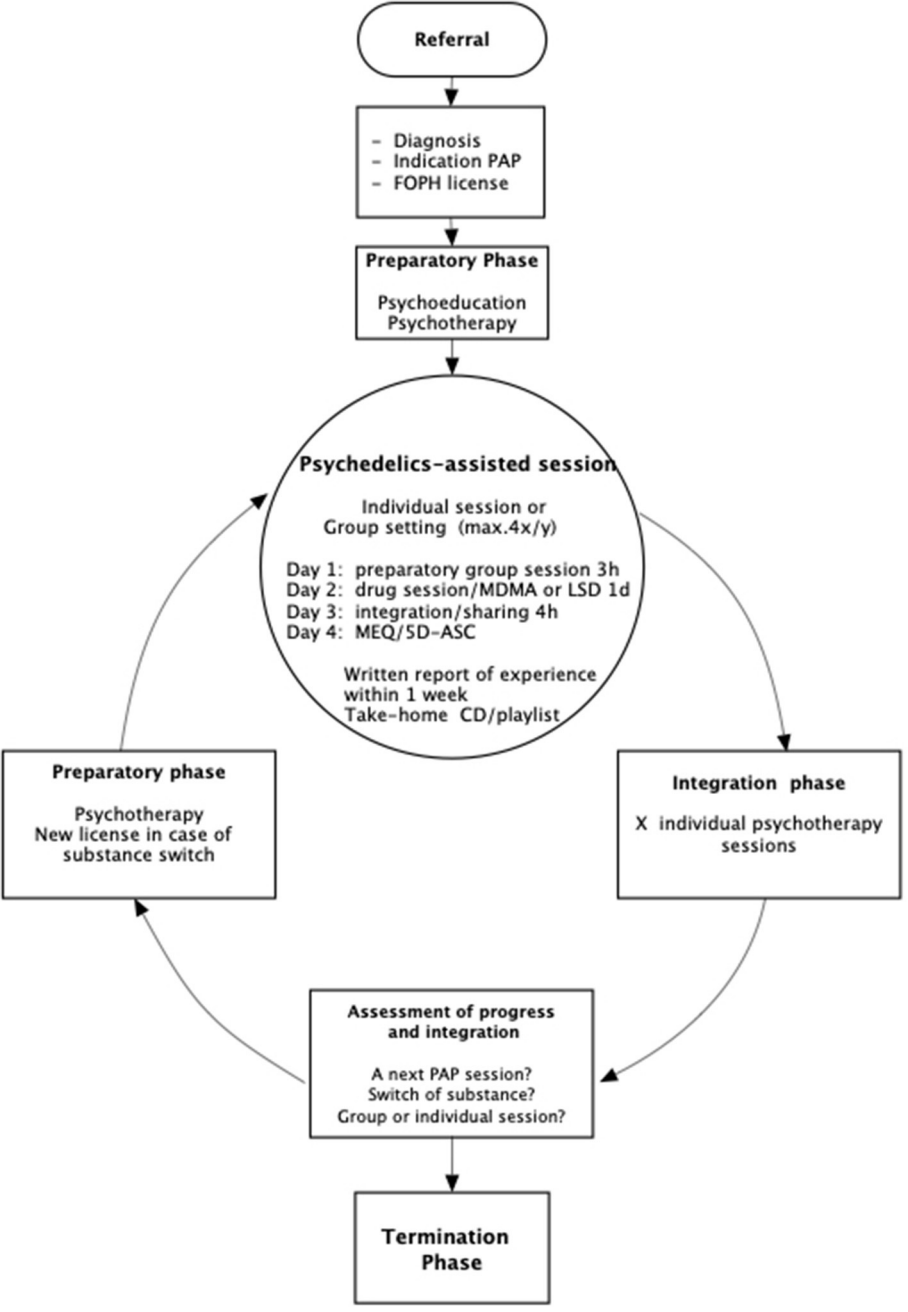
Flow-chart of procedures.

Figure 1 shows the procedures of the treatment. Depending on the diagnosis, expectations and intentions, the anticipated need for individual support during the substance experience and the degree of group competence, patients were assigned directly to the group setting or to a series of individual substance–assisted sessions before joining to the group. Among the diagnoses, cluster headache, end-of-life issues, or ASD were treated primarily in individual settings.

Reasons for first starting with individual sessions were a strong fear of the psychedelic experience itself or also pronounced fear of the group situation of having to express oneself in front of strangers. A further frequent objection to the group was observed in the presence of a severe shame issues, as is quite common in cases of sexual trauma. Even when participants regularly attended the group it could happen that in extraordinary therapeutic situations individual psychedelic-assisted sessions were added. The intervals between psychedelic-assisted sessions varied substantially, depending on the therapeutic process and the pace of integration as well as other factors such as professional obligations that could not be postponed. Only a small number of patients attended all available group sessions.

Before a patient engaged in trauma-focused psychedelic-assisted psychotherapy, the following steps were necessary:

a) Psychoeducation: Conveying a comprehensive map of consciousness, how humans react acutely and chronically to trauma, the nature of psychedelic-assisted psychotherapy, risks and benefits and what patients can expect from the therapist and from psychedelics-assisted psychotherapy.
b) Establishing a therapeutic relationship and alliance, which from the perspective of the therapist is stable enough to be able to support the patient during and after the anticipated phases of emotionally charged confrontation with traumatic material occasioned by the psychedelic experience.
c) Learning basic skills for awareness and regulation of emotions, e.g., self-soothing and breathing techniques, as well as for ensuring personal safety.
d) Formulating a tentative working hypothesis on how symptoms developed and are related to the biography and the trauma history.

### Group Procedures

The psychedelic-assisted group sessions took place four times a year (2–4 months apart, depending on holidays and availability of the therapists) in the group room of the private practice of the first author located in a quiet residential area on the outskirts of a small town in Switzerland. The dates of the sessions were scheduled in advance and were communicated to the participants. Two separate rooms were available in case someone felt too exposed while in the group, urgently needed to talk with one of the therapists, or felt overstimulated and wanted to retreat for a while. The location offered the possibility to go outside and experience nature if desired. The number of participants was limited to twelve, with three therapists of which one was female. A larger group would have overstrained the resources of the therapists to appropriately guide the group and support participants in their individual process and also unduly limit time for individual preparation before the session as well as during the sharing after the psychedelic experience.

Because the MDMA experience is of shorter duration than with LSD and because some participants would become talkative, restless or wished to interact with others under the influence of MDMA, there was a need to structure and guide the group experience so that participants experiencing LSD were not disturbed or distracted too much. During the first 4–5 h of the experience we therefore advised the participants to refrain from direct interactions with other group members in order to not disturb the unfolding and deepening of the inner process, i.e., mystical-type peak experience in the opening and plateau phases of the experience.

From one session to the next, the two authors consistently alternated in the role of main guide. The main guide was responsible for the decoration of the room (candles, flowers, etc.), the moderation of group discussions, the selection and playing of music, as well as for giving general instructions and guiding the group during the substance experience. The two other therapists acted as co-therapists/sitters, supporting anyone who required or requested help or attention.

A 3-day format was used for the LSD or MDMA experience. Participants met in the evening before the psychedelic experience for 3 h beginning with warm-up exercises such as breath or body awareness exercises. This was followed by the presentation of new group members and the discussion of recent developments, problems, expectations, concerns and fears of the participants as well as setting of intentions for the next day. Participants were encouraged to interact with each other in the discussion. The therapists finally answered questions about dosage, effects and procedures of the LSD/MDMA experience and also gave instructions on how to appropriately manage the altered state of consciousness and cope with difficulties such as anxiety or distressing physical/emotional states and/or memories. The participants then decided where in the room and beside whom they wanted to spend the following day and accordingly arranged their places.

Participants reclined on mattresses in a circle, and armchairs were available if, for example, participants were aged, disabled, or had trouble getting up from the floor. Some patients brought various meaningful personal belongings, souvenirs or photographs, which were arranged in the middle of the circle. The evening closed with a piece of music. Participants spent the night in the group room, at home or at a local B&B or hotel if they came from out of town.

On the day of the psychedelic experience, the group would meet at 9.30 a.m. Last minute questions and concerns were addressed and/or short contributions were made, e.g., sharing a dream. The ingestion of the substances took place at about 10 a.m. To shorten the time until the onset of effects and to reduce anxiety (which was always present in the group at the beginning) the main guide repeated instructions or suggested some body awareness exercises.

Participants were advised to keep their eyes closed as much as possible and to direct attention to their inner process. We did not use blindfolds, but these were available on request. It was therefore up to the participants to learn to guide and focus attention, similarly to many meditation traditions. Music and silence/stillness alternated.

Music is an invaluable and essential ingredient of most forms of psychedelic-assisted psychotherapy, and holds the potential to touch and influence the psyche beyond the conscious and verbal-cognitive dimension (31–33). We always played music over speakers in order to achieve a collective listening experience. Sometimes participants brought music of their choice to be played during the experience. The therapists also played the frame drum, gong or handpan. Live music enhanced effects by adding a personal musician-listener dimension to the experience.

The music we played was carefully selected according to the phase of the experience, the atmosphere of the moment in the group, the flavor, rhythm and message of the music and lyrics as well as its intended effects (evocative, calming, activating, confrontational, etc.). We played music which to the participants was new and unknown, carefully monitoring its immediate effects on the group and the individuals. Table 4 presents a typical music playlist. In total, music was played for about one third of the session, while two-thirds of the process took place in silence.

Stillness is considered a tool of confrontation and is at the same time a typical (blissful) feature of the mystical-type peak experience (34).

The therapists monitored, moved around the room, and sat with participants spontaneously and on request. We checked on their process but avoided lengthy verbal exchanges. If there were no issues, we sat quietly with them. If a problem arose, participants were verbally instructed on how to manage the difficult situation, offering touch (holding hands or putting a hand on a tense or painful spot of the body), thus helping the patient to overcome avoidance and resistance, and redirecting their awareness to the therapeutic process.

Vital signs (e.g., blood pressure, temperature, etc.) were monitored only in exceptional circumstances, when necessary, or when the clinical situation was unclear (e.g., severe headaches or unclear pain symptoms).

About 6–7 h after ingestion of the substance and depending on the extent to which its effects had worn off, we invited the participants to express how they were feeling and to give short statements concerning what they had experienced so far. This frequently helped participants who were stuck or distressed and confused about their experience to relax, re-orient and start to cognitively reflect and integrate the experience.

At about 6 p.m. we served a light dinner in the form of a picnic, sitting together on the floor of the group room. At 7 p.m. we closed the group session and participants could then stay together for the rest of the evening without the therapists, talk together or listen to music, go for a walk or go home. We then drove people to wherever they were staying for the night.

On the final day, the group met again at 9.30 a.m. We began by listening to a piece of music played the previous day, in order to help participants tune in to their experience again. All participants were then invited to share their experience in detail. This integrative talking session was considered of utmost importance, aimed at helping the participants verbalize the rich, blissful, ineffable and yet often overwhelming, difficult and painful experience, which reflected their psychodynamic and spiritual process.

Verbalizing is an important and crucial step toward understanding and integration. It is also an invaluable opportunity for verbal therapeutic interventions as the participants were still in a very open, non-defensive and responsive mental and emotional state. The aspect of group dynamics on the day after was also more prominent and visible. Experiencing feedback and compassion from other group members further facilitated and potentiated the individual integration process. The integration talking session took about 4 h and terminated with final instructions for the following time on what do in case of an emotional crisis, sudden unexpected recall of additional traumatic material, confusing thoughts, etc. We once again ended by listening to some music together and then tidying up the room.

All participants were required to write a comprehensive report of the experience and fill out the 5D-ASC (5 Dimensions Altered States of Consciousness) and the MEQ (Mystical Experience Questionnaire) questionnaires. The reports were discussed in the regular integrative psychotherapy. Reports and questionnaires were subject to an accompanying study approved by the FOPH examining reported acute drug effects (35).

Participants received a take-home CD or a list of the tracks that were played. Listening to the music later helped to resolve momentary difficult emotional situations and was helpful in the further integration process.

## Results

### Development of the Group as a Whole

From 2014 to 2020, 22 group sessions took place. Initially, patients with trauma-related disorders, as well as OCD, depression, cluster headache, anxiety disorders in conjunction with Parkinson's disease and multiple sclerosis were included in the group. We quickly realized that for the forging of a stable working alliance within the group, the individual goals diverged too much. Participants with trauma-related disorders, anxiety disorders and depression, already primed by previous psychotherapies, implicitly accepted that this therapy required a lengthier individual and group therapeutic process which involved questioning themselves, revisiting traumatic events and engaging in a process with the group, whereas participants with other diagnoses, e.g., with cluster headache, had more short-term goals such as quick symptom reduction. Moreover, cluster-headache treatment with psychedelics is considered as more of a pharmacological than a psychotherapeutic treatment.

Over time a core group of participants who continuously attended psychedelic-assisted sessions was formed. They also began to meet and provide mutual support outside of the therapy, and new friendships developed. The more advanced they were in their individual long-term therapeutic process the more they engaged directly in supporting and confronting their peers during the sessions in a co-therapeutic sense, as well as taking care of and serving as a model for newcomers, helping to experience what secure attachment, mutual responsibility and care for each other really mean.

As in every group, conflicts later arose, for example, from transference. We usually advised that the conflict in the group be declared before the experience, in order to focus on one's own part of the conflict and to try to resolve the conflict with the person involved during the last phase of the experience or during the sharing on the 3rd day, when participants are more open, compassionate and less defensive.

Another observation was that specific issues would manifest and spread across the group, such as a specific emotion like fear or a focus on relationships to important figures of childhood, which would then be utilized therapeutically. More experienced participants were generally more conscious of and open to the group process, and would express their perceptions spontaneously, by saying for example “I perceive so much anger in the group…” Preconscious conflicts and ineffable childhood dramas tended to be re-enacted in the group. These kinds of group dynamics are not specific to psychedelic-assisted groups, but can be enhanced considerably under the influence of psychedelics.

### Typical Individual Developments

#### Treatment Modalities and Outcomes

Table 2 shows that there was a large spread in treatment modalities between the majority of the 50 patients attending only group sessions (35 of 50), and those who combined group and individual setting (nine of 50). Only six out of 50 patients were treated solely in an individual setting. Some reasons for adding individual sessions were momentary excessive demands in an intense therapeutic phase, or the wish to explore the issue of fear of closeness to others.

**Table 2:** Treatment modalities.

**Treatment modalities**	PG^**^	PO^**^	**Total**
Termination of treatment with psychedelics after 1–3 sessions^*^	10 (38%)	5 (21%)	15 (30%)
Only group setting	17 (56%)	18 (75%)	35 (70%)
Only individual setting	5 (19%)	1 (4%)	6 (12%)
Group and individual setting combined	4 (15%)	5 (21%)	9 (18%)
Only MDMA	3 (12%)	3 (13%)	6 (12%)
Only LSD	18 (69%)	8 (33%)	26 (52%)
MDMA and LSD	5 (19%)	13 (54%)	18 (36%)
Mean number of applications/patient: MDMA only subgroup (range: 1–4)	2.5	2.4	-
Mean number of applications/patient: LSD only subgroup (range: 1–5)	4.1	3.6	-
Mean number of applications/patient: MDMA/ and LSD subgroup (range MDMA: 1–9; range LSD: 1–12)	6.7/6.7	4.3/5	-

* *Reasons for early termination were: marked and swift symptom reduction/remission, also compliance problems, difficult first psychedelic experiences and consequently reluctance to continue treatment, other reasons not related to therapy*.

** *PG, Peter Gasser; PO, Peter Oehen*.

The fact that Peter Gasser treated more patients with LSD is simply due on one hand to the fact that his patient pool contained more cases with anxiety disorders and cluster headache/migraine cases, for which psychedelic treatment was initiated directly with LSD, and on the other hand that he was more familiar and comfortable with LSD. Peter Oehen's patient pool, on the contrary, contained more trauma-associated cases, with MDMA being the primary indication based on current data.

The most frequent diagnosis being c-PTSD, we were able to distinguish a typical pattern of the therapeutic process in this subpopulation. Despite optimal preparation, the first psychedelic-assisted sessions were usually very trying for all involved. In the initial MDMA experiences most patients experienced first a short phase with positive and pleasant MDMA effects followed by a confrontational phase with direct recall of traumatic material and intense negative emotions. In cases of dissociative amnesia for the trauma, the recollection of the trauma often began with somatic symptoms such as pain, cramps, paraesthesia, etc., often located in the traumatized areas of the body, then moving on to sensory perceptions e.g., sounds, smells, and/or fragmentary images and memories.

A complete process of recollection of trauma could range from one to a lengthy series of psychedelic-assisted sessions depending on how early in life the traumatization took place and how extensive it was. In cases of severe dissociative states or non-epileptic seizures, these tended to be activated first and then needed special attention to help patients reorient and to gradually tolerate the underlying emotional distress. It was only then that the recollection and reprocessing of trauma would begin.

In parallel or with some delay, the dysfunctional relationship patterns and attachment problems emerged and became a second crucial focus of therapy.

Another observation concerned the fact that after a certain number of sessions, patients kept reliving similar traumatic events without gaining any further benefit from this confrontation (“looping”). At this point, they were guided to shift attention away from these memories and to focus more on the here and now of their lives, i.e., on resolving the trauma sequelae. This was also a reason for switching from MDMA to LSD. In general, we made the observation that the occurrence of the typical positive mystical type experience (MTE) led to more favorable post-session developments. In patients with more pronounced treatment needs such MTE were only observed in later phases of therapy. Even without the occurrence of MTE, these patients made substantial progress.

Table 3 presents the outcomes of the 50 reported psychedelic-assisted therapies at the end of the reporting period. Outcomes were determined by clinical judgment, since scales such as the HAMD (Hamilton depression scale) or the CAPS (clinician administered PTSD scale) were not used regularly and are therefore not reported here. In the cases of depression, OCD and anxiety disorders of symptoms according to ICD-10 were assessed. In the two ASD cases, the degree of social anxiety was assessed.

**Table 3:** Outcomes as assessed by clinical judgement in regard to overall symptom reduction and general well-being.

**Nr. Patients**	**Index diagnosis**	**Deteriorated**	**Not improved**	**Clinically improved**	**Remission**	**Regular termination of psychotherapy**	**Dropout/ irregular termination of treatment**	**Continuation of psychotherapy**
Outcomes PO^*^								
15 (63%)	Trauma-related disorders	0	2 (13%)	9 (60%)	4 (27%)	8 (53%)	1 (7%)	6 (40%)
1 (4%)	Anxiety disorders	0	0	1 (100%)	0	0	0	1 (100%)
6 (25%)	Depression	0	2 (34%)	2 (33%)	2 (33%)	5 (83%)	0	1 (17%)
1 (4%)	ASD	0	0	1 (100%)	0	1 (100%)	0	0
1 (4%)	OCD	0	1 (100%)	0	0	0	1 (100%)	0
Outcomes PG*								
6 (23%)	Trauma-related disorders	0	2 (33%)	4 (67%)	0	2 (33%)	0	4 (67%)
9 (35%)	Anxiety Disorders	0	3 (33%)	4 (45%)	2 (22%)	3 (33%)	1 (11%)	5 (56%)
4 (15%)	Depression	0	2 (50%)	2 (50%)	0	2 (50%)	1 (25%)	1 (25%)
1 (4%)	ASD	0	1 (100%)	0	0	0	0	1 (100%)
1 (4%)	OCD	0	0	0	1 (100%)	1 (100%)	0	0
5 (19%)	Cluster headache	1 (20%)	3 (60%)	1 (20%)	0	4 (80%)	1 (20%)	0

* *PO, Peter Oehen; PG, Peter Gasser*.

**Table 4:** Typical music playlist.

**Title**	**Artist**	**Album**	**Length**
Earth & Sky	Cye Wood & Lisa Gerrard	The Trail of Genghis Khan	5:39
Words of Amber	Ólafur Arnalds	Ólafur Arnalds	3:23
The Shores Of Loch Brann/Hazafelé (Homeward)	Márta Sebestyén	The Best Of Márta Sebestyén	4:35
It Diede (You Never Know)	Mari Boine	Eight Seasons/Gávcci Jahkejudgu	4:40
Night	Ludovico Einaudi	Elements (Deluxe)	5:31
Dream 13 (Minus Even)	Max Richter	From Sleeo	8:53
Song Of The Birds	Caroline Dale	Such Sweet Thunder	3:26
Empires Of Light	Caroline Dale	Such Sweet Thunder	4:16
Song of Good Hope	Glen Hansard	Rhythm And Repose	3:48
Mother's Wingspan	Ben Leinbach	The Spirit of Yoga	18:54
Sangen Om Fyret Ved Tornehamn	Kari Bremnes	Svarta bjørn	4:38
Ipmiliin háleasteapmi / Conversation With God	Mari Boine	An Introduction to	4:41
Dans Le Silence	Martha Wainwright	Trauma: Chansons de la série télé Saison #4	3:24
Nursery Rhyme Of Innocence And Experience	Natalie Merchant	Leave Your Sleep [Disc 1]	5:11
Khabar Kana	Souad Massi	Ô Houria	3:54
Le Bonheur Il Suffit D'Une Phrase	Lokua Kanza	Toyebi Te	3:25
All Related	Nessi Gomes	Diamonds & Demons	6:09
Cuatro Vientos	Danit	Aliento	7:27
In the End	Passenger	The Boy Who Cried Wolf	3:05
Love Song	Marianne Faithfull	Horses and High Heels	4:38

In the case of trauma-related disorders, the major symptom clusters were assessed to determine the outcome (see paragraph “complex post-traumatic disorder”). All five symptom clusters of C-PTSD were attenuated. The clearest improvements were enhanced emotion regulation and relational capacities, as well as a decrease of intrusive and dissociative symptoms. Continued improvement was observed with each session. Overall, of the 21 patients with trauma-related disorders, four achieved remission, 13 had improved and three did not improve. Two of ten cases with anxiety disorders remitted, five cases improved clinically, and three cases did not improve. The OCD subgroup showed one case with remission and one non-improved case. The cluster headache subgroup contained only on case of improvement in contrast to three cases with no improvement and unfortunately one case with a deteroration (see below). In the ASD subgroup one individual improved and the other did not improve.

At the end of the reporting period, 20 of 50 participants were still in ongoing (partly psychedelic-assisted) psychotherapy, with further reductions of symptoms and improvement of wellbeing.

#### Depression

Pre-existing antidepressant medication was a problem for which there was not yet a good or consistent solution available. On one hand, short-term tapering as we used in our method, has been shown to diminish response to MDMA-assisted therapy (36). On the other hand, lengthier taper periods risk a relapse of depression prior to the psychedelic session. Another minor problem was the discontinuation syndrome: about half of the patients showed mild to moderate symptoms for a few days. In two cases, the taper period had to be extended to 4 weeks because symptoms were not tolerated well. The tapering was therefore adjusted on a case-by-case basis.

In two cases, the dose of the antidepressant medication was lowered but not stopped (escitalopram from 20 to 10 mg and venlafaxine from 75 to 37.5 mg) because of these difficulties. Both patients reported having experienced “a strange trip,” meaning that they felt uncomfortably emotionally numb or detached during their psychedelic experience. Furthermore, no obvious relationship between prior antidepressant medication and improvement was noticed.

Patients with a depressive disorder (10 cases) or with a comorbid chronic/recurrent depression to c-PTSD (in 12 cases) who were under treatment with antidepressants until the psychedelic experience and who then discontinued medications tended to relapse after MDMA although the therapy and trauma processing progressed. Especially in the beginning of a relapse of depression following a substance experience it was hard to discern “normal” but emotionally difficult and trying trauma processing from symptoms associated with depression. This pattern was observed more after MDMA and much less after LSD. We therefore resorted to reinstalling the antidepressant medication immediately after sessions, or as soon as a relapse of depression was suspected. The relapse of depression usually took place in a time span of 3–6 weeks. After two or three such episodes, individual cues could be identified which allowed for the timely reinstallation of antidepressant medication.

We observed that comorbid depression to trauma-related disorders tended to improve (four out of twelve) or remit completely (eight out of twelve) in later phases of therapy. Antidepressants could be stopped in five cases and had to be continued in two cases (total of seven cases with pre-existing antidepressant medication).

In depression as index diagnosis, two of 10 cases achieved remission, four of 10 cases improved clinically, and four of 10 cases did not improve at all.

#### Adverse Events, Difficult Situations, Discontinuation of Treatment, and Conflicts in Group

One 48-year-old woman showed a prolonged delusional phase after LSD in the context of an especially painful and confusing episode of childhood sexual abuse, which resolved during the integrative psychotherapy, requiring a limited medication with quetiapine 25–50 mg for sleep.

Another 38-year old woman suffering severely from cluster headache for more than 10 years attended one group session with LSD 100 mcg. She had used high doses of all kinds of analgesics, tryptanes, and ketamine nasal spray for acute headache attacks. During this session, she developed an acute severe headache attack that was successfully treated with ketamine and lorazepam. She felt disappointed and refused to repeat the LSD experience.

During the period of acute drug effects during the session, nobody tried to leave the premises. Some patients requested to sit outside on the balcony of the group room for a certain time or wanted to be alone in the annex room. We had several confrontations with heavy smokers who wanted to go outdoors to smoke during the psychedelic experience, which had not been part of the agreement or group rules. It usually turned out that the urge to smoke was related to something unpleasant happening inside which they wanted to avoid by smoking. Leaving the group room all too frequently for snacks or restroom visits were similar avoidance patterns we observed. Another avoidance pattern that needed to be addressed was when patients sat up, started walking around, started obsessively taking notes, etc. Chattering, which distracted fellow participants was a minor but frequent problem that also needed to be addressed.

Patients with comorbid borderline traits tended to have more difficult psychedelic experiences that were emotionally more intense, overwhelming and distressing. These required more support from the therapists. Interestingly, they also received a lot of understanding, compassion and often “co-therapeutic support” (e.g., hugging, hand-holding) from fellow patients. Until the emotional regulation capacity improved, the integration phase in the weeks afterwards was often similarly turbulent and erratic, requiring close follow-up. Suicidal thoughts and mild self-harm were reported several times, but no suicide attempts happened. It was possible to manage these situations within the usual framework of integrative psychotherapy.

Unforeseen early termination of treatment happened in two cases where patients did not comply with the agreements of the treatment and did not engage appropriately in the integrative psychotherapy. Another reason was in one case a first difficult psychedelic experience and consecutive reluctance to continue this form of treatment. One patient discontinued the treatment unexpectedly following a (transference) conflict with one of the therapists, which could not be addressed adequately.

The most common conflicts between group participants involved disruptive behavior (loud chatter, careless, or importunate interactions with fellow patients) during the experience or when someone was overly indirect during the sharing on the 3rd day, avoiding talking in a personal and authentic manner about their experience.

#### Case Vignette

In 2013, following previous depressive episodes, 51-year-old Anne came in for treatment for severe major depression with suicidality. She had followed continuous treatment with antidepressants for many years. She worked in the health profession, was married and had two adolescent children. At the age of 16 she had begun to feel unhappy, emotionally numb and repeatedly fell into dissociative states. At 20 years she began the first of three extensive courses of psychotherapy. In summary, the psychotherapies stabilized her in everyday life, reconnected her to her emotions and improved body awareness. However, her traumatic childhood remained unaddressed because of marked avoidance.

Unwanted at birth, Anne grew up as the first of three children in a very frosty family atmosphere. Her mother denied her children all physical and emotional contact, was chronically depressed and ailing, and had put the blame solely on Anne for the latter. The only nurturing physical contact she experienced was with her sister with whom she shared a bedroom. The two sisters consoled each other by cuddling and singing together in bed at night. Her father was a sadistic, hot-tempered, violent and unpredictable man who enjoyed inflicting pain and fear upon his children. Anne remembered him almost drowning her several times during swimming lessons and molesting her in the bathroom. She also remembered her father sneaking into their bedroom at night and abusing her sister sexually, leaving her completely helpless and increasing her feelings of guilt for being unable to prevent the abuse. Even today Anne still feels responsible for her sister who consequently developed a severe and disabling BPD and eating disorder in her adolescence. The repetitive witnessing of her sister being sexually abused together with the prolonged physical and emotional neglect and the massive guilt conflict led to deeply rooted emotional and relational problems, negative self-perception and low self-esteem which had troubled her since adolescence. She was chronically distrustful, shunned conflicts when personally involved, was not aware of her personal boundaries and let others take advantage of her out of chronic feelings of guilt and shame. Above all, she was unable to regulate her emotions and impulses appropriately, hurting herself when she was emotionally overwhelmed.

She was subsequently diagnosed with chronic depression, including a current severe episode as well as moderate degree complex PTSD. Her depression wore off only slowly under pharmacological treatment with venlafaxine 150 mg/daily. At the same time, she had great difficulties speaking in therapy about her family of origin, repetitively dissociating or going numb when addressing traumatic memories. In this phase we were able to outline her relational and emotional problems, her chronic intrusive and dissociative symptoms and enhance her awareness of the constant distress and avoidance patterns that she had been experiencing for decades, as well as developing a map of the traumatic events and conditions in her childhood.

At this point and after being in intensive psychotherapy for more than 1 year she had her first MDMA experience in a group setting. She labeled it an “emotional breakthrough” and apart from experiencing positive and negative emotions of previously unknown intensity, she felt great relief about at last being able to be certain that the abuse of her sister had really happened and acknowledging that it was not her fault that her father had abused her sister. She was now able to speak freely about the abuse despite her father's lifelong tabooing and threats. Depression did relapse after the first MDMA experiences but no longer required medication. The therapeutic alliance was instantly freed of distrust and reluctance. Consequently, nightmares of her father coming into the bedroom at night frightened her increasingly and gradually the suspicion of having been abused herself dawned on her. This was painfully confirmed by the following MDMA experiences. It took eight MDMA sessions to complete the narrative of her own abuse. At the same time, she opened up emotionally in relationships, gaining trust and self-esteem.

In the group, she befriended a fellow patient and developed a sister-transference to her. The processing of this transference reaction was very important to overcome her chronic feelings of guilt toward her biological sister and to gain independence, self-worth and authenticity. This led to her being able to interact with her fellow patient (and the others) in a good and increasingly mature way, with clear boundaries.

In contrast, she chronically needed reassurance and confirmation of her perceptions and behavior in a childlike manner for a long time during the integrative therapy. Minimal uncertainties and misunderstandings would destabilize and depress her again and again. She also began to worry about what went wrong in early infancy and in the relationship to her mother. For this reason and because the therapy stalled at this point, we switched from MDMA to LSD (in a dose of 150 mcg). In the following four LSD experiences different aspects of the sexual abuse already covered in the MDMA experiences were reviewed and revisited in a much more confrontative, detailed and relentless manner. More importantly, she was able to identify and explore lifelong attachment problems, which turned out to have originated perinatally. Intense feelings of pain, despair, disgust, shame, guilt, abandonment and insecurity burdened her again. Then, after an impressive and overwhelming death-rebirth experience under LSD she was able to change the abovementioned patterns in a short time, abandon her habitual negative self-perception and become increasingly self-confident, assertive, more independent, self-determined and resilient. Earlier, after the first few MDMA experiences, she had already come to terms with her father who suffered from dementia and was able to forgive him. After experiencing LSD, her relationship to her mother improved to the point where her mother admitted her mistakes and failures, making reconciliation possible and enabling closeness to her mother for the first time. Needless to say, the relationship to her husband and children—a secondary focus of the therapy—matured and deepened during this phase. This most recent development allowed for the thinning out of the integrative psychotherapy and the initiation of termination phase of the psychedelic-assisted psychotherapy.

At termination of the treatment, she made the following statement when asked about the significance of the group setting for her treatment:

“*I was a very lonely and disconnected person before I joined the group. The psychedelic group experience helped me to open up, to be close to others, to express myself, to become authentic and to build relationships. I realized that other people also had severe problems and to listen to them and watch them change from one session to the next was fascinating, enlightening and encouraging. To talk to the group about all the bad and painful things that had happened to me as a child was an extremely helpful and healing experience. I appreciated the group rules and the clear structure of the workshops. The preparation evening before and the sharing and integration the morning after the experience were very important parts of the treatment.”*

#### Comment

The course of Anne's psychedelic-assisted psychotherapy includes all typical aspects of effective trauma-focused therapy: an extensive psychoeducational preparation phase, followed by a lengthy and intense drug-initiated phase of recall and processing leading to a coherent trauma narrative, simultaneously targeting difficult trauma-related emotions and enhancing emotional regulation skills, as well as initiating cognitive restructuring, changes in self-perception and meaning. Parallel to these central focuses, relational problems with significant others were addressed and resolved.

One important difference to non-drug-assisted trauma-focused psychotherapy is that the therapeutic relationship became acutely less ambivalent, more stress-relieved, closer and resilient once MDMA was involved. MDMA also increased the change motivation, perseverance and the emotional window of tolerance, as well as decreasing the degree of post-traumatic avoidance substantially. In Anne's case, MDMA alone was not able to address and resolve the early life traumatization together with the corresponding dysfunctional patterns of behavior and self-perception resulting from the maternal rejection, neglect and induction of guilt. MDMA paved the way with the processing of childhood trauma, while LSD addressed the deeply rooted relational, attachment and self-relational problems.

## Discussion

Based on modern psychodynamic and psychotraumatological concepts, as well as on the preliminary empirical evidence for psychedelic-assisted psychotherapy, a model of group psychedelic-assisted psychotherapy for a population of patients with a variety of diagnoses is presented, highlighting the treatment of treatment-refractory post-traumatic disorders in which MDMA and LSD were used sequentially. Doses and intervals were adapted to the individual needs and the therapeutic process.

The treatment of c-PTSD required larger numbers of psychedelic experiences than the MDMA-assisted treatment of single trauma that is currently being investigated in phase 3 studies (16). We saw a wide range of one to nine MDMA applications and one to 12 LSD applications. Considering that such disorders often began early in life and had a significant impact on personality development, this is not surprising. A small number of patients (four) with comorbid borderline traits were also treated but, as expected, they required additional attention and support.

The clinical results for the subgroup of participants with trauma-related disorders were more favorable than in the other subgroups (depression, anxiety disorders).

We also had the impression that a part of the problem of non-improvement or relapse of depression could be due to a more difficult transfer of the insights and psychedelic experiences into everyday life than seen in other disorders such as anxiety disorders.

Administering LSD and MDMA in the same group required careful dosing of the substances, as well as consistent structuring and guiding of the group process. We observed the group setting to be more challenging and confrontative than the individual setting by expanding the psychedelic experience with a collective dimension. In our experience, group psychedelic experiences intensified and deepened the individual therapeutic process and made it therapeutically more efficient in regard to outcomes than in an individual setting. The group setting also allowed us to treat more patients in the given time.

There were no safety problems and no serious adverse events requiring hospitalization.

The psychedelic group therapy model presented here suggests the feasibility and safety of this approach. Given the current high need for more effective treatments for chronic trauma-related disorders, OCD, chronic depression and anxiety disorders, the “restricted medical use program” is an invaluable way to help otherwise treatment-resistant patients until these psychedelics have become registered drugs.

Several limitations exist: the outcomes presented here are purely descriptive and based solely on clinical judgement without using standard scales to document improvements, and therefore of very limited significance. Because of the nature and the legal constraints of the “restricted medical use program,” we were not able to establish a prospective and controlled research design.

Despite this, the practical experiences that were made with this program can possibly provide directions for future research, the training of future psychedelic therapists and the practical application of these substances once they have been registered as prescription drugs.

## Data Availability Statement

The original contributions presented in the study are included in the article/supplementary material, further inquiries can be directed to the corresponding author.

## Ethics Statement

Ethical review and approval was not required for the study on human participants in accordance with the local legislation and institutional requirements. The patients/participants provided their written informed consent to participate in this study. Written informed consent was obtained from the individual(s) for the publication of any potentially identifiable images or data included in this article.

## Author Contributions

PO wrote the first draft of the manuscript. Both authors contributed to the article and approved the submitted version.

## Funding

Open access publication was paid by the Swiss Medical Society for Psycholytic Therapy (SAePT).

## Conflict of Interest

Both authors work in private psychiatric practices in Switzerland. PG is president of the Swiss Medical Society for Psycholytic Therapy and has counseling agreements with CompassPathways, MindMed, and Reconnect labs.

## Publisher's Note

All claims expressed in this article are solely those of the authors and do not necessarily represent those of their affiliated organizations, or those of the publisher, the editors and the reviewers. Any product that may be evaluated in this article, or claim that may be made by its manufacturer, is not guaranteed or endorsed by the publisher.

## Footnotes

^1^https://www.admin.ch/opc/en/classified-compilation/19981989/index.html#a8a (accessed July 9, 2018).
